# Prevention of Intra-Abdominal Adhesion by Bi-Layer Electrospun Membrane

**DOI:** 10.3390/ijms140611861

**Published:** 2013-06-04

**Authors:** Shichao Jiang, Wei Wang, Hede Yan, Cunyi Fan

**Affiliations:** Department of Orthopaedics, Shanghai Sixth People′s Hospital, Shanghai Jiaotong University School of Medicine, 600 Yishan Road, Shanghai 200233, China; E-Mails: mailjsc@163.com (S.J.); wahaa1987@163.com (W.W.); yanhede@hotmail.com (H.Y.)

**Keywords:** postoperative adhesion, hyaluronic acid, electrospun, barrier, adhesion prevention

## Abstract

The aim of this study was to compare the anti-adhesion efficacy of a bi-layer electrospun fibrous membrane consisting of hyaluronic acid-loaded poly(ɛ-caprolactone) (PCL) fibrous membrane as the inner layer and PCL fibrous membrane as the outer layer with a single-layer PCL electrospun fibrous membrane in a rat cecum abrasion model. The rat model utilized a cecal abrasion and abdominal wall insult surgical protocol. The bi-layer and PCL membranes were applied between the cecum and the abdominal wall, respectively. Control animals did not receive any treatment. After postoperative day 14, a visual semiquantitative grading scale was used to grade the extent of adhesion. Histological analysis was performed to reveal the features of adhesion tissues. Bi-layer membrane treated animals showed significantly lower adhesion scores than control animals (*p* < 0.05) and a lower adhesion score compared with the PCL membrane. Histological analysis of the bi-layer membrane treated rat rarely demonstrated tissue adhesion while that of the PCL membrane treated rat and control rat showed loose and dense adhesion tissues, respectively. Bi-layer membrane can efficiently prevent adhesion formation in abdominal cavity and showed a significantly decreased adhesion tissue formation compared with the control.

## 1. Introduction

Intra-abdominal adhesions are common complications following abdominal surgery and entail chronic abdominal pain and intestinal obstructions [1]. In about 10% of adhesion cases, the adhesion tissue may act as a band for the bowel to twist around, and thus lead to tissue ischemia and eventual necrosis [2]. Furthermore, the fibrous tissue increases the difficulty of the secondary surgery. Consequently, numerous investigators have focused on the physical barriers in preventing postoperative adhesion in abdominal cavity [3–5]. However, issues of efficacy and practicality make these barriers remain daunting challenges for surgeons.

Fibrinolysis is a key factor in intra-abdominal adhesion prevention [6]. Thereafter, enhanced fibrin degradation is a common concept to reduce the adhesions. Recently, various agents such as fibrinolytic agents, anticoagulants, and anti-inflammatory agents have been employed to minimize fibrin formation and promote its degradation [3,7,8]. However, these agents alone did not prevent adhesion formation effectively because of their short-term residence. Furthermore, many of these agents have the reported wound healing inhibition effects [9]. Therefore, there is still a need for improved means to prevent intra-abdominal adhesions formation.

Hyaluronic acid (HA) has the anti-adhesive property that shields the damaged peritoneal surfaces in the development of intra-abdominal adhesions as well as having a beneficial effect on wound healing [10,11]. Furthermore, the application of hyaluronan-based agents to reduce intra-abdominal adhesions and to modulate the fibrinolysis give a promising therapy [12,13]. In previous studies, HA gel was used in an attempt to reduce tissue adhesion, but it was ideal because of its high rheological behavior and rapid degradation [14]. Furthermore, when made or loaded into an anti-adhesion film such as Seprafilm, HA may make the barrier too brittle and difficult to apply [15]. With the development of crosslinking method, some crosslinking modified hyaluronic acid hydrogel films were synthesized. By changing the concentration of crosslinking agents and hyaluronic acid, the swelling ratios and storage modulus are improved, and better stability and mechanical characterization are obtained [16]. Recently, a bi-layer electrospun fibrous membrane, containing a HA-loaded PCL membrane as inner layer, has been fabricated. The results indicate that with the increase of HA content, the tensile strengths of the bi-layer fiber membranes were decreased, while the tensile modulus were increased. So when using the bi-layer fiber membranes with proper HA content, we can obtain the bi-layer fiber membranes which have better mechanical characterization, hydrophilicity and flexibility together compared with crosslinked hyaluronic acid hydrogel films [17]. This may be an advancement of the bi-layer membrane. However, the *in vivo* anti-adhesion effect in abdominal cavity has not been recently reported.

In this study, we hypothesized that the bi-layer membrane can efficiently prevent adhesion formation in abdominal cavity. To verify our hypothesis, we fabricate the bi-layer electrospun fibrous membrane by co-electrospinning of HA and PCL and sequential electrospinning of a HA-loaded PCL inner layer and a PCL outer layer and then the *in vivo* anti-adhesion efficacy of the bi-layer membrane was investigated in a rat cecum abrasion model.

## 2. Results and Discussion

### 2.1. Characterization of Bi-Layer Electrospun Fibrous Membrane

**Figure 1:**
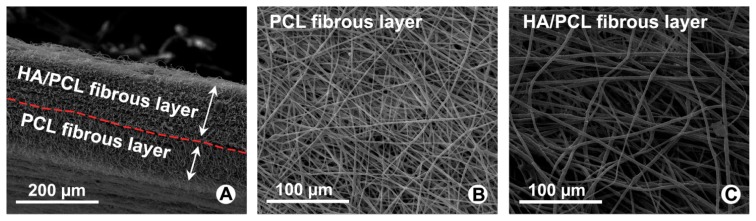
SEM observation for cross-sectional and surface morphological features of Polycaprolactone (PCL) fibers and hyaluronan (HA)/PCL fibers. (**A**) Cross-sectional of bi-layer electrospun fibrous membrane; (**B**) PCL electrospun fibers of outer layer; (**C**) HA/PCL electrospun fibers of inner layer.

From cross-sectional morphology of Figure 1A we can learn that thicknesses of the bi-layer membrane were 150 μm for inner HA-loaded PCL layer and 80 μm for outer PCL layer. Both surface morphology of the bi-layer electrospun fibrous membrane were shown in Figure 1B,C. The fibers are uniform in size, randomly interconnected structures and seemingly smooth with no bead in the fibrous structure.

### 2.2. Gross Observation

**Figure 2:**
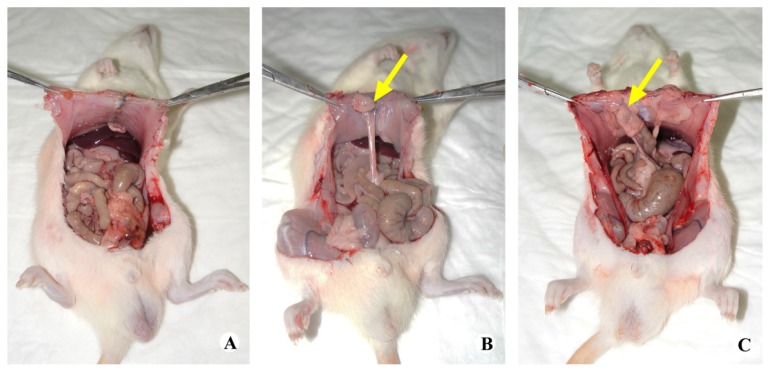
Gross evaluation of a rat cecum abrasion model after 14 days. (**A**) Group treated with bi-layer membrane; (**B**) Group treated with single-layer membrane; (**C**) Untreated group. Yellow arrows indicate the adhesion areas.

**Figure 3:**
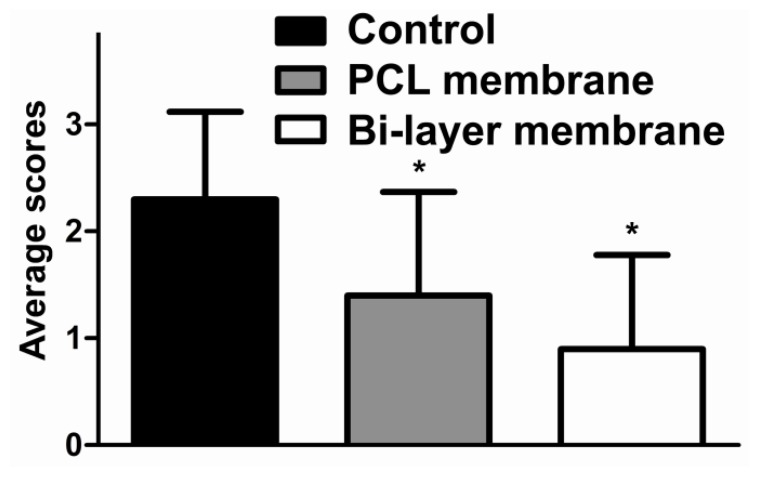
The average scores of intra-abdominal adhesion in the control group, bi-layer membrane group and PCL electrospun fibrous membranes group are presented as mean ± SD (*n* = 8).(* *p* < 0.05 *vs*. Control).

After 14 days, the tissue adhesion extent was graded and averaged after the surgical exploration of the injured sites (Figure 2). Both bi-layer and single-layer membranes reduced tissue adhesion. In the animals treated with bi-layer electrospun fibrous membrane, adhesions formations were rarely revealed between the cecum and the abdominal wall. The application of bi-layer membrane was better for anti-adhesion than the PCL membrane. All the animals without any treatments of their peritoneal defects showed large and severe adhesions which required to be separated by sharp dissection. According to the extent of intra-abdominal adhesion score from macroscopic observations, the average scores in the bi-layer membrane group were lower than the control groups (*p* < 0.05) (Figure 3). Animals treated with PCL membrane also demonstrated a lower adhesion score (*p* < 0.05) than the control. There was no statistical difference between the two treatments, but the bi-layer membrane showed a slight trend towards a lower score than the PCL membrane (*p* = 0.220).

### 2.3. Histological Assessments

**Figure 4:**
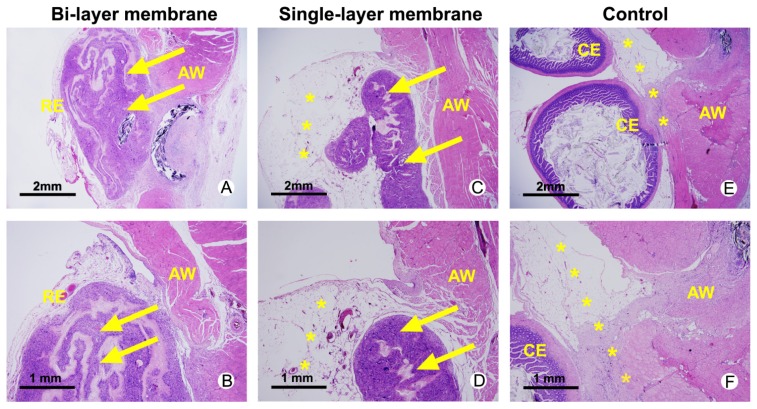
Hematoxylin and eosin (HE) staining of abdominal wall defects from bi-layer membrane treated (**A**,**B**), single-layer membrane treated (**C**,**D**) and untreated animals with severe adhesion (*) between the abdominal wall (AW) and the cecum (CE) surface. Remnants of the materials are highlighted with yellow arrows and located inside the re-epithelialized (RE) abdominal wall defect site.

Histological analysis of rats receiving different treatment was performed in Figure 4. The anti-adhesion membranes treated animals had visible remnants of biomaterials after 14 d. The bi-layer membrane treated specimen displayed re-epithelialized abdominal wall defect site on the remnants of biomaterials with no adhesions between the cecum and abdominal wall surfaces. Loose bundles of fibrous tissue formation in the group wrapped with single-layer membrane and re-epithelialized abdominal wall defect site is unclear. Views of the abdominal wall and cecum surface in untreated group displayed a strong, fibrous tissue layer formed between the cecum and abdominal wall surfaces.

### 2.4. Discussion

In this study, we fabricated a HA/PCL inner layer by co-electrospinning of HA and PCL solution and subsequently fabricated a bi-layer electrospun fibrous membrane by sequential electrospinning of PCL as outer layer as previous study [15], and thus the proper physical property and the ability of controlled releasing of HA have been investigated. From *in vivo* results of the intra-abdominal adhesion, we can learn that adhesion tissue can be prevented by the electrospun fibrous membrane in abdominal cavity. From whether gross observation or histological assessments, we can observe that the extent of adhesion in the bi-layer membrane group and PCL membrane group were lower than the control groups. Meanwhile, re-epithelialized abdominal wall was observed, this result suggested that the electrospun fibrous membrane could improve wound healing. Furthermore, the bi-layer electrospun fibrous membrane demonstrated a better anti-adhesion effect than PCL membrane in abdominal cavity of rat specimens.

Abdominal adhesions can be regulated by the plasminogen system. If the fibrinolysis of these intra-abdominal fibrin deposits fails, the fibrin matrix may serve as a scaffold for fibroblasts and capillary in growth and for extracellular matrix deposition. Then, the adhesions will become fibrous [18]. Hyaluronan-based agents have been reported to reduce adhesion formation after surgery by reducing the abscess formation in experimental peritonitis [12]. The efficient anti-adhesion effect was supported by the gross view (Figure 2) and histological assessments (Figure 4) in this study. Possible mechanisms of this action include physical barrier of wound surfaces, promotion of peritoneal repair, modulation of the inflammatory response and enhanced fibrinolysis. Thereafter, HA can reduce intra-abdominal adhesions by modulation of the fibrinolysis. However, the exact mechanism needs further study.

Optimally, materials for anti-adhesion and regenerative medicine applications should degrade over the course of tissue regeneration to allow complete repair by host tissue, so controlled release of HA is also a critical aspect of this study, In the past, rapid clearance of HA from the peritoneal cavity remained major challenges, but with the development of new chemically modified methods, like *in situ* cross-linkable hydrogel system [19], double crosslinking strategy [20], glycidyl methacrylate modified HA [21] *et al.*, the time of degradation and controlled release has been prolonged and can be depended on the concentration of the gel components, but their high rheological behavior and lower mechanical strength limits the clinical application.

The bi-layer electrospun fibrous membrane would be expected to act as a better HA delivery system. First, compared with HA-loaded PCL membranes, the high rheological behavior and lower mechanical strength of the HA gels has been demonstrated. Second, HA-loaded PCL membranes which has better controlled release and biocompatibility has been indicated in previous study. Last, if HA was mixed into a single-layer barrier, the tensile force may decrease as the HA content increases. Seprafilm (Genzyme Corporation, Cambridge, MA, USA) consisting of sodium hyaluronate was approved for abdominal surgeries as a type of degradable barrier in the US [22]. Upon deployment, it isolated injured tissue to prevent adhesion formation and simultaneously benefited the damaged tissue to heal [23,24]. However, these films are brittle and thus relatively difficult to apply. Furthermore, they aggressively adhered to any moisture on the surgeon′s gloves during placement; thus, their use is limited primarily to open surgical procedures [15]. In contrast, better tensile strength of the bi-layer system than single-layer fibrous membranes loaded with HA has been certificated.

In this study, the bi-layer membrane was developed to gain better *in vivo* anti-adhesive effect than PCL membrane. The outer PCL layer was prepared to play the anti-adhesion roles, namely to maintain the anti-adhesion effect to prevent intra-abdominal adhesion from surrounding repaired tissue. The novel inner HA/PCL layer of this bi-layer membrane can efficiently prevent adhesion formation, possibly due to the ability of HA to modulate fibrinolysis. Based on our successful result of the macroscopic and histological evaluation, we can learn that the bi-layer membrane can prevent adhesion formation in abdominal cavity and the inner layer may even promote the formation of the re-epithelialized abdominal wall. However, the promotion of formation of re-epithelialized abdominal wall needs further study.

As an *in vivo* anti-adhesion result, the bi-layer electrospun fibrous membrane demonstrated a better anti-adhesion effect than PCL membrane in abdominal cavity of rat. This is possibly due to the synergistic effect of biological function of HA and physical barrier of bi-layer membrane. Previously, PCL film has been tried to prevent intra-abdominal adhesion and it led to fewer adhesions than Seprafilm in rat adhesion model [25]. Thereafter, including the improper mechanical property of Seprafilm, this is another one reason why Seprafilm was not directly used as a control in this study.

Our study just demonstrates a preliminary success of the biomimetic bi-layer sheath membrane in a two week *in vivo* study, the side-effects from administration of this adhesion barrier, like foreign body reaction, intra-peritoneal abscess formation was not our focus in this study. This will be the next plan of our future studies to investigate the later effects on intra-abdominal adhesions after the complete degradation of the biomimetic sheath.

Even if this bi-layer membrane is effective in reducing mainly the extent of adhesions in animal models, it should be stressed that its efficacy remains to be proved in clinic further.

## 3. Experimental Section

### 3.1. Preparation of Bi-Layer Electrospun Fibrous Membrane

Co-electrospinning and sequential electrospinning were used in this study. Briefly, 1.0 g Polycaprolactone (PCL, Mw = 50 kDa, Mw/Mn = 1.6, Jinan Daigang Co., Jinan, China) was added into 3.0 g tetrahydrofuran (THF, GuoYao Regents Co., Shanghai, China) while 1.0 g hyaluronan (HA, sodium salt, Mw = 1.0 MDa, Yuancheng Thecnolgy Co., Wuhan, China) was completely dissolved in 15.0 g H_2_O. Then, HA solution were mixed with the PCL/THF solution in weight ratios 0:100 (pure PCL) and 12:100, and then 1g hexafluoroisopropanol (HFIP, Guo Yao Regents Co., Shanghai, China) was added into solution. Grounded aluminum foil was used as the collector. The inner layer of the bi-layer electrospun fibrous membrane was first fabricated by microgel of HA and PCL solution for co-electrospinning and then a PCL outer layer was added by sequential electrospinning. Finally, the bi-layer membranes were collected on the surface of a roll of aluminum foil and vacuum dried at room temperature for 24 h.

### 3.2. Rat Cecum Abrasion Surgical Model

The procedures and handing of the animals were performed accordance to the guidelines of Shanghai Jiao Tong University School of Medicine and the National Institutes of Health. Rats (Sprague-Dawley, weight: 350 to 450 g) were used to assess the anti-adhesion potential of bi-layer electrospun fibrous membrane in abdominal cavity. Animals were anesthetized with intramuscular injection of ketamine (20 mg/kg). Defects (1 × 1 cm) in the left abdominal wall and the cecum were created respectively by removing the peritoneum through a mid-line laparotomy incision [26]. After immersing the bi-layer electrospun fibrous membranes in phosphate buffered saline (PBS) for a few minutes, they were applied to each wound before the abdomen was closed. For comparison, a group of animals was treated with PCL electrospun fibrous membranes. Control animals did not receive any intervention. The wound was closed using 4–0 silk sutures layer by layer.

### 3.3. Macroscopic Evaluation

Before sacrificing the animals, signs of inflammation or ulcer were visually checked on the skin incision. Then, the animals were sacrificed after 14 days postsurgery. To evaluate the extent of adhesion, a semiquantitative grading scale was scored at the repair site on the basis of the macro views: grade 0, no adhesions; grade 1, thin and filmy adhesions easily separable with blunt dissection; grade 2, moderate adhesions with freely dissection plane; grade 3, severe with fibrosis difficult dissection plane [27–29]. Intra-abdominal adhesions were evaluated by two independent investigators blinded to treatment.

### 3.4. Histological Evaluation

Tissues were dissected, rinsed in PBS, and fixed overnight in 4% paraformaldehyde buffered with PBS. After dehydration with increasing concentrations of ethanol and the preserved tissues were embedded in paraffin. Then, 4-mm-thick transverse sections were cut and stained by hematoxylin and eosin (HE) to observe the morphology of the tissues.

### 3.5. Statistical Analysis

The adhesion was evaluated on eight animals for each sample, and the results are expressed as mean ± standard deviation. Statistical software SPSS 10.0 (Chicago, IL, USA) was used to analyze the data by one-way analysis of variance; *p* < 0.05 considered significant.

## 4. Conclusions

In conclusion, bi-layer membrane can efficiently prevent adhesion formation in abdominal cavity and showed a significantly decreased adhesion tissue formation compared with the control. It could provide surgeons with another easily-applied choice to prevent abdominal adhesions.
